# *HNF1B* and Endometrial Cancer Risk: Results from the PAGE study

**DOI:** 10.1371/journal.pone.0030390

**Published:** 2012-01-27

**Authors:** Veronica Wendy Setiawan, Jeffrey Haessler, Fredrick Schumacher, Michele L. Cote, Ewa Deelman, Megan D. Fesinmeyer, Brian E. Henderson, Rebecca D. Jackson, Jens-S Vöckler, Lynne R. Wilkens, Shagufta Yasmeen, Christopher A. Haiman, Ulrike Peters, Loïc Le Marchand, Charles Kooperberg

**Affiliations:** 1 Department of Preventive Medicine, Keck School of Medicine, University of Southern California, Los Angeles, California, United States of America; 2 Division of Public Health Sciences, Fred Hutchinson Cancer Research Center, Seattle, Washington, United States of America; 3 Department of Oncology, Wayne State University School of Medicine and Population Studies and Disparities Research, Karmanos Cancer Institute, Detroit, Michigan, United States of America; 4 Information Sciences Institute, University of Southern California, Marina Del Rey, California, United States of America; 5 Center for Clinical and Translational Science, The Ohio State University, Columbus, Ohio, United States of America; 6 Epidemiology Program, University of Hawaii Cancer Center, Honolulu, Hawaii, United States of America; 7 Department of Obstetrics and Gynecology, University of California Davis, Davis, California, United States of America; Fondazione Istituto FIRC di Oncologia Molecolare (IFOM), Italy

## Abstract

We examined the association between *HNF1B* variants identified in a recent genome-wide association study and endometrial cancer in two large case-control studies nested in prospective cohorts: the Multiethnic Cohort Study (MEC) and the Women's Health Initiative (WHI) as part of the Population Architecture using Genomics and Epidemiology (PAGE) study. A total of 1,357 incident cases of invasive endometrial cancer and 7,609 controls were included in the analysis (MEC: 426 cases/3,854 controls; WHI: 931cases/3,755 controls). The majority of women in the WHI were European American, while the MEC included sizable numbers of African Americans, Japanese and Latinos. We estimated the odds ratios (ORs) per allele and 95% confidence intervals (CIs) of each SNP using unconditional logistic regression adjusting for age, body mass index, and four principal components of ancestry informative markers. The combined ORs were estimated using fixed effect models. Rs4430796 and rs7501939 were associated with endometrial cancer risk in MEC and WHI with no heterogeneity observed across racial/ethnic groups (P≥0.21) or between studies (P≥0.70). The OR_per allele_ was 0.82 (95% CI: 0.75, 0.89; P = 5.63×10^−6^) for rs4430796 (*G* allele) and 0.79 (95% CI: 0.73, 0.87; P = 3.77×10^−7^) for rs7501939 (*A* allele). The associations with the risk of Type I and Type II tumors were similar (P≥0.19). Adjustment for additional endometrial cancer risk factors such as parity, oral contraceptive use, menopausal hormone use, and smoking status had little effect on the results. In conclusion, *HNF1B* SNPs are associated with risk of endometrial cancer and that the associated relative risks are similar for Type I and Type II tumors.

## Introduction

Endometrial cancer is the most common gynecological cancer in developed countries. A recent genome-wide association study (GWAS) identified common single nucleotide polymorphisms (SNPs) in *HNF1B* associated with endometrial cancer risk in women of European background [1]. The same SNPs, rs4430796 and rs7501939, are also associated with prostate cancer [2] and type 2 diabetes [3], [4]. We examined the association between these SNPs and risk of endometrial cancer in two large prospective cohort studies with comprehensive risk factor data: the Multiethnic Cohort Study (MEC) and the Women's Health Initiative (WHI), as part of the Population Architecture using Genomics and Epidemiology (PAGE) study [5]. We also examined the associations between *HNF1B* and endometrial cancer across racial/ethnic groups and tumor histological types, and effect modification by known endometrial cancer risk factors.

## Materials and Methods

PAGE is an ancillary study to both WHI and MEC, and has been approved by the WHI and MEC steering committees. The PIs for the PAGE studies within WHI and MEC have further authority for analyses within the scope of the original applications.

### Study population

PAGE study is a National Human Genome Research Institute (NHGRI)-supported collaboration with a primary focus of deep characterization of well-replicated genetic risk variants identified in GWAS and their relationships to various phenotypes and traits (e.g., lipids, diabetes, heart disease, cancers) in diverse epidemiologic studies. Included in the characterization process is 1) replication of the original association in a population of similar genetic ancestry as the original GWAS, 2) generalization of the association to diverse populations such as African Americans, Asians, Hispanic/Mexican Americans, and other groups, 3) identification of gene-environment interactions, and 4) identification of pleiotropy. The details of PAGE design and methods have been presented by Matise et al [5]. The PAGE study samples were drawn from four large population-based studies or consortia [5]; however, the current analysis only included women from the MEC and the WHI. The MEC is a prospective cohort study consisting of 215,251 adult men and women living in Hawaii and California predominantly from five populations: European American, African American, Native Hawaiian, Japanese, and Latino (Hispanic/Mexican Americans) [6]. A subset of cohort participants (∼70,000) has available DNA samples. Incident cases of endometrial cancer were identified through cohort linkage to the population-based cancer Surveillance, Epidemiology, and End Results (SEER) registries in California and Hawaii. Controls were selected from female cohort participants without a self-reported hysterectomy at baseline and who were free of cancer as at December 31, 2008. Controls were individually matched to cases based on age at cohort entry, race/ethnicity, and study area (Hawaii or California). The MEC endometrial cancer case-control study included 426 invasive endometrial cancer cases and 3,854 controls. The WHI is comprised of an observational study and four clinical trials covering the components of dietary modification, hormone therapy, separately for women with and without a uterus, and supplementation of calcium/vitamin D [7]. The study consists of 161,808 postmenopausal women from various racial/ethnic groups. Incident cases of endometrial cancer in the cohort were identified through self-report, which was ascertained at least annually and confirmed by clinicians after reviewing the pathology reports [8]. Controls were selected from cohort participants without a self-reported hysterectomy at baseline and who were free of cancer through September 1, 2009. Controls were individually matched to cases based on age at baseline, date of enrollment, race/ethnicity, and trial arms. The WHI endometrial cancer case-control study included 931 invasive endometrial cancer cases and 3,755 controls.

### Tumor histology

We used the International Classification of Diseases for Oncology (ICD-O-3) code to classify endometrial cancer cases as Type I or Type II [9]–[11]. Unopposed estrogens are suspected to affect Type I but not Type II tumors [12]. Type I included endometrioid (ICD-O-3 code: 8380, 8381, 8382, 8383), adenocarcinoma tubular (8210, 8211), papillary adenocarcinoma (8260, 8262, 8263), adenocarcinoma with squamous metaplasia (8570), mucinous adenocarcinoma (8480, 8481), and adenocarcinoma NOS (8140). Type II included clear cell (8310), serous (8441), papillary serous (8460, 8461), squamous cell (8050, 8070, 8071, 8072), adenosquamous (8560), small cell carcinoma (8041), and mixed cell adenocarcinoma (8323). Cases with a sarcoma diagnosis were not included in the analysis.

### SNP selection and genotyping

The two *HNF1B* SNPs (rs4430796 and rs7501939) were part of 167 (MEC) and 183 (WHI) well-replicated genetic risk variants identified from GWAS genotyped in the PAGE study to explore pleiotropic effects on several cancer sites. Genotyping was performed using the TaqMan Open Array Genotyping System (Life Technologies/Applied Biosystems) as part of the PAGE initiative. The average genotype completion rate was 98.0% in the MEC and 99.9% in the WHI. The concordance of blinded duplicates was 99.7% in the MEC and 99.5% in the WHI. Hardy-Weinberg Equilibrium (HWE) for each allele was assessed in each racial/ethnic group in controls; no deviation from HWE was observed (at the P<0.01 level) across more than one racial/ethnic group, suggesting that such deviations are likely due to chance and not to genotyping error.

### Statistical analysis

Known risk factors for endometrial cancer (*i.e.* parity, oral contraceptive use, menopausal hormone use, smoking status, and diabetes status were obtained from the baseline questionnaire data. Per allele odds ratios (ORs) and 95% confidence intervals (CIs) for the SNP-endometrial cancer association were calculated using unconditional logistic regression. Models were adjusted for age (continuous), body mass index (BMI) (<25, 25-<30, ≥30 kg/m^2^), and the top four ancestry principal components. Principal components derived from >100 ancestry informative markers were estimated using the EIGENSTRAT method [13]. Parity, oral contraceptive use, menopausal hormone use, smoking status, and diabetes status were considered as potential confounders. Test of interaction with race/ethnicity and potential effect modification by endometrial cancer risk factors was assessed using log-likelihood test statistics comparing models with and without the interaction term (cross product between the SNP and race/ethnicity or risk factor of interest). The combined ORs and 95% CIs were estimated from each study's OR using a fixed effects model and between-study heterogeneity was examined using the Q test statistics. We used polytomous logistic regression to calculate ORs and 95% CIs for Type I and Type II endometrial cancer. All racial/ethnic groups were included in this subgroup analysis. All P values are two-sided.

## Results

The characteristics of cases and controls in the MEC and the WHI are shown in Table 1. The mean ages of cases and controls were similar in each study. The majority of women in the WHI were European American (93.2% of cases and 80.3% of controls); there were very few Asian/Pacific Islander (n = 8) and Latino (n = 20) cases. The MEC included sizable proportions of women from other racial/ethnic groups: 20.5% African American, 30.3% Japanese, and 18.7% Latino. Compared to controls, cases were heavier, more likely to have fewer births, and to be diabetic. Cases were less likely to have used OCs or to have ever smoked.

**Table 1:** Characteristics of Cases and Controls in the Multiethnic Cohort Study (MEC) and the Women's Health Initiative Study (WHI).

	MEC		WHI	
	CasesN = 426	ControlsN = 3854	CasesN = 931	ControlsN = 3755
Mean age^1^ (SD)	65.6 (8.3)	66.2 (8.8)	63.7 (7.0)	64.6 (7.4)
Race/ethnicity, n (%)				
European American	106 (24.9)	813 (21.1)	868 (93.2)	3037 (80.3)
African American	68 (16.0)	820 (21.3)	35 (3.8)	350 (9.2)
Hawaiian	27 (6.3)	344 (8.9)		
Asian^2^/Pacific Islander	121 (28.4)	1204 (31.2)	8 (0.9)	161 (4.3)
Latino	104 (24.4)	673 (17.5)	20 (2.1)	207 (5.5)
Body mass index (kg/m^2^), n (%)				
<25	146 (34.3)	1792 (46.5)	306 (32.9)	1411 (37.6)
25-<30	113 (26.5)	1220 (31.7)	253 (27.2)	1293 (34.4)
≥ 30	163 (38.3)	796 (20.7)	364 (39.1)	1018 (27.1)
Missing	4 (0.9)	46 (1.2)	8 (0.9)	33 (0.9)
Parity, n (%)				
Nulliparous	73 (17.1)	454 (11.8)	149 (16.0)	509 (13.6)
1–2	150 (35.2)	1313 (34.1)	322 (34.6)	1213 (32.3)
3–4	144 (33.8)	1405 (36.5)	360 (38.7)	1390 (37.0)
≥5	56 (13.1)	648 (16.8)	97 (10.4)	632 (16.8)
Missing	3 (0.7)	34 (0.9)	3 (0.3)	11 (0.3)
Oral contraceptive use, n (%)				
Never	238 (55.9)	2006 (52.1)	576 (61.9)	2368 (63.1)
Ever	178 (41.8)	1766 (45.8)	355 (38.1)	1387 (36.9)
Missing	10 (2.4)	82 (2.1)		
Menopausal hormone use, n (%)				
Never	252 (59.2)	1877 (48.7)	361 (38.8)	2196 (58.5)
Past	65 (15.3)	578 (15.0)	124 (13.3)	539 (14.4)
Current	94 (22.0)	1258 (32.6)	444 (47.7)	1018 (27.1)
Missing	15 (3.5)	141 (3.7)	2 (0.2)	2 (0.1)
Smoking status, n (%)				
Never	263 (61.7)	2209 (57.3)	486 (52.2)	1956 (52.1)
Past	131 (30.8)	1139 (29.6)	395 (42.4)	1490 (39.7)
Current	27 (6.3)	458 (11.9)	40 (4.3)	264 (7.0)
Missing	5 (1.2)	48 (1.3)	10 (1.1)	45 (1.2)
Diabetes, n (%)				
No	388 (91.1)	3556 (92.3)	887 (95.3)	3610 (96.1)
Yes	38 (8.9)	298 (7.7)	44 (4.7)	143 (3.8)
Missing				2 (0.1)

1 Age at diagnosis for cases and age at blood draw for controls in the MEC; age at baseline for cases and controls in the WHI.

2 Japanese in the MEC, approximately 25% Chinese, 50% Japanese, and 25% other groups in the WHI.

We found that rs4430796 and rs7501939 were associated with risk of endometrial cancer in European Americans in the MEC and the WHI (Table 2). The combined OR_per allele_ was 0.83 (95% CI: 0.75, 0.92; P = 4.00×10^−4^) for rs4430796 (*G* allele) and 0.79 (95% CI: 0.71, 0.88; P = 1.30×10^−5^) for rs7501939 (*A* allele). No heterogeneity between studies was observed (P≥0.59). The rs4430796 and rs7501939 were in strong linkage disequilibrium (LD) in our European-American controls (r^2^ = 0.61 in the MEC; r^2^ = 0.66 in the WHI).

**Table 2:** Association between *HNF1B* variants and endometrial cancer.

			rs4430796 (*A/G*)			rs7501939 (*G/A*)		
Race/ethnicity	Study	Number of cases/controls	Allele Frequency Cases/Controls	OR^1^ (95% CI)	P-value	Allele Frequency Cases/Controls	OR^1^ (95% CI)	P-value
European American	MEC	106/813	0.45/0.51	0.79 (0.59, 1.05)	0.11	0.34/0.41	0.73 (0.53, 0.99)	0.045
	WHI	868/3037	0.45/0.49	0.84 (0.75, 0.93)	0.0015	0.36/0.41	0.80 (0.72, 0.90)	0.00015
	Combined^3^			0.83 (0.75, 0.92)	4.00×10^−4^		0.79 (0.71, 0.88)	1.30×10^−5^
African American	MEC	68/820	0.61/0.64	0.80 (0.55, 1.16)	0.23	0.48/0.51	0.88 (0.61, 1.26)	0.47
	WHI	35/350	0.59/0.65	0.81 (0.49, 1.35)	0.41	0.41/0.52	0.61 (0.35, 1.02)	0.065
	Combined^3^			0.80 (0.59, 1.09)	0.15		0.78 (0.58, 1.06)	0.11
Asian/Pacific Islander	MEC	121/1204	0.31/0.38	0.74 (0.55, 0.99)	0.045	0.27/0.33	0.76 (0.56, 1.04)	0.09
	WHI	8/161	0.38/0.29	1.44 (0.48, 4.12)	0.49	0.38/0.26	1.76 (0.55, 5.56)	0.32
	Combined^3^			0.78 (0.58, 1.03)	0.078		0.80 (0.60, 1.08)	0.15
Latino	MEC	104/673	0.38/0.41	0.83 (0.60, 1.16)	0.28	0.31/0.34	0.85 (0.60, 1.22)	0.39
	WHI	20/207	0.30/0.48	0.42 (0.19, 0.84)	0.02	0.18/0.41	0.29 (0.11, 0.65)	0.006
	Combined^3^			0.74 (0.55, 1.00)	0.052		0.73 (0.53, 1.02)	0.065
Hawaiian	MEC	27/344	0.33/0.34	0.80 (0.41, 1.59)	0.53	0.31/0.30	0.87 (0.43, 1.74)	0.69
All groups^2^	MEC	426/3854	0.41/0.46	0.80 (0.69, 0.93)	0.0048	0.33/0.38	0.80 (0.68, 0.94)	0.0068
	WHI	931/3755	0.45/0.50	0.83 (0.75, 0.92)	0.00059	0.36/0.41	0.79 (0.71, 0.88)	1.87×10^−5^
	Combined^3^			0.82 (0.75, 0.89)	5.63×10^−6^		0.79 (0.73, 0.87)	3.77×10^−7^

1 Odds ratio per allele obtained from logistic regression adjusting for age (continuous), 4 ancestry principal components, BMI (<25, 25-<30, ≥30 kg/m^2^).

2 P interaction with race/ethnicity in the MEC ≥0.63; P interaction with race/ethnicity in the WHI ≥0.21;

3 Combined ORs were calculated using a fixed effects model.

In the MEC, consistent associations were observed in African Americans, Hawaiians, Japanese and Latinos, i.e. reduced risk associated with the *G* allele of rs4430796 or with the *A* allele of rs7501939 (Table 2). There were limited numbers of non-European descent women in the WHI, especially the Asian/Pacific Islander group (8 cases and 161 controls). In African Americans and Latinos, we observed consistent associations with those observed among European Americans. No evidence was observed of heterogeneity in the ORs by race/ethnicity (P≥0.21). Combining the MEC and the WHI results, the OR_per allele_ ranged between 0.74 and 0.80 for rs4430796 and between 0.73 and 0.80 for rs7501939 in African Americans, Asians/Pacific Islanders, and Latinos. The two SNPs were in high LD in Asians (r^2^ = 0.80) and Latinos (r^2^ = 0.65) and in lower LD in African Americans (r^2^ = 0.33).

In the analysis of all race/ethnicity groups combined, the OR_per allele_ for rs4430796 was 0.80 (95% CI: 0.69, 0.93; P = 0.0048) and 0.83 (95% CI: 0.75, 0.92; P = 0.00059) in the MEC and the WHI, respectively (Table 2). The all groups' OR_per allele_ for rs7501939 was 0.80 (95% CI: 0.68, 0.94; P = 0.0068) and 0.79 (95% CI: 0.71, 0.88; P = 1.87×10^−5^) in the MEC and the WHI, respectively. When we combined the results from the MEC and the WHI, the OR_per allele_ was 0.82 (95% CI: 0.75, 0.89; P = 5.63×10^−6^) for rs4430796 and 0.79 (95% CI: 0.73, 0.87; P = 3.77×10^−7^) for rs7501939. No heterogeneity between studies was observed (P≥0.70). Further adjustment for parity, oral contraceptive use, menopausal hormone use, smoking status, diabetes status and clinical trial participation (dietary modification, hormone therapy, or observational study) for the WHI had little effect on the results.

The associations of *HNF1B* SNPs with Type I and Type II tumors are shown in Table 3. In both studies, rs4430796 and rs7501939 were significantly associated with Type I tumors. Both SNPs were also associated with reduced risk of Type II tumors, but the association was only significant for rs4430796 in the MEC. No evidence of heterogeneity between studies was observed (P≥0.18). The combined OR_per allele_ for rs4430796 was 0.83 (95% CI: 0.76, 0.90; P = 2.79×10^−5^) for Type I tumors and 0.78 (95% CI: 0.61, 0.99; P = 0.041) for Type II tumors. The combined OR_per allele_ for rs7501939 was 0.80 (95% CI: 0.73, 0.87; P = 1.00×10^−6^) for Type I tumors and 0.75 (95% CI: 0.58, 0.95; P = 0.020) for Type II tumors. Neither study found significant differences between the associations of *HNF1B* SNPs with Type I and Type II tumors (P≥0.19 in the MEC; P≥0.80 in the WHI).

**Table 3:** Association between *HNF1B* variants and Type I and Type II endometrial cancer.

			rs4430796 (*A/G*)			rs7501939 (*G/A*)		
Tumor type	Study	Number of cases/controls	Allele Frequency Cases/Controls	OR^1^ (95% CI)	P-value	Allele Frequency Cases/Controls	OR^1^ (95% CI)	P-value
Type I	MEC	354/3854	0.41/0.46	0.82 (0.69, 0.94)	0.020	0.33/0.38	0.81 (0.68, 0.97)	0.019
	WHI	837/3755	0.45/0.50	0.83 (0.74, 0.92)	0.00073	0.36/0.41	0.79 (0.71, 0.88)	4.45×10^−5^
	Combined^2^			0.83 (0.76, 0.90)	2.79×10^−5^		0.80 (0.73, 0.87)	1.00×10^−6^
Type II	MEC	45/3854	0.37/0.46	0.59 (0.37, 0.94)	0.025	0.32/0.38	0.66 (0.41, 1.07)	0.093
	WHI	101/3755	0.47/0.50	0.86 (0.65, 1.15)	0.31	0.36/0.41	0.78 (0.58, 1.03)	0.093
	Combined^2^			0.78 (0.61, 0.99)	0.041		0.75 (0.58, 0.95)	0.020

1 Odds ratio per allele obtained using polytomous logistic regression adjusting for age (continuous), 4 ancestry principal components, and BMI (<25, 25-<30, ≥30 kg/m^2^).

2 Combined ORs were calculated using a fixed effects model.

To determine whether the associations of *HNF1B* variants and endometrial cancer were influenced by diabetes, we examined the OR for the SNP-endometrial cancer relationship among diabetics and non-diabetics separately (Table 4). Significant associations were observed only among non-diabetics in both studies. In the WHI, the test for interaction was statistically significant for rs4430796 (P = 0.028) and borderline significant for rs7501939 (P = 0.054). No significant interaction was observed in the MEC.

**Table 4:** Association between *HNF1B* variants and endometrial cancer by diabetes status.

			rs4430796 (*A/G*)		rs7501939 (*G/A*)	
Diabetes Status	Study	Number of cases/controls	Allele Frequency Cases/Controls	OR^1^ (95% CI)	Allele Frequency Cases/Controls	OR^1^ (95% CI)
Non-diabetic	MEC	388/3556	0.41/0.46	0.80 (0.69, 0.94)	0.33/0.38	0.80 (0.68, 0.94)
	WHI	887/3610	0.45/0.50	0.81 (0.73, 0.91)	0.35/0.41	0.77 (0.69, 0.86)
	Combined^2^			0.81 (0.74, 0.88)		0.78 (0.71, 0.85)
Diabetic	MEC	38/298	0.42/0.47	0.76 (0.44, 1.30)	0.36/0.38	0.90 (0.52, 1.57)
	WHI	44/143	0.57/0.49	1.41 (0.85, 2.37)	0.49/0.43	1.37 (0.80, 2.37)
	Combined^2^			1.05 (0.73, 1.53)		1.11 (0.76, 1.64)

1 Odds ratio per allele obtained from logistic regression adjusting for age (continuous), 4 ancestry principal components and BMI.

2 Combined ORs were calculated using a fixed effects model.

Test for interaction was assessed using log-likelihood test statistics comparing models with and without the interaction term.

P interaction for rs4430796 was 0.028 (WHI) and 0.93 (MEC); P interaction for rs7501939 was 0.054 (WHI) and 0.58 (MEC).

We also examined effect modification of the association between *HNF1B* SNPs and endometrial cancer by BMI, parity, OC use, menopausal hormone use and smoking status (Table S1 and S2) and found no significant interaction.

## Discussion

We show that the *HNF1B* SNPs (rs4430796 and rs7501939) identified in a recent endometrial cancer GWAS [1] are associated with endometrial cancer risk in two independent studies and that the associations were observed across multiple racial/ethnic groups. We also show that similar associations are seen for both Type I and Type II tumors and across all categories of BMI, parity, OC use, menopausal hormone use and smoking status.

The risk estimates observed among European Americans this study (OR_rs4430796_ = 0.83; OR_rs7501939_ = 0.79) were similar to those reported by the initial GWAS (OR_rs4430796_ = 0.84; OR_rs7501939_ = 0.85) [1]; the most significant SNP in the GWAS (rs4430796) however was not the most strongly associated SNP in this study, which underlies the fact that neither SNP is the causal SNP.

*HNF1B* (formerly known as *TCF2*) is a transcription factor that encodes three isoforms: isoforms A and B which act as transcriptional activators and isoform C which acts as a transcriptional repressor [14]. Rare mutations in *HNF1B* have been associated with maturity-onset diabetes of the young subtype 5 (MODY5), renal cysts, pancreatic atrophy, and uterine abnormalities caused by incomplete Mullerian duct fusion and Mullerian duct aplasia [15], [16]. Differential expression of *HNF1B* has been associated with prostate cancer recurrence [17] and differential expression of *HNF1B* isoforms has been found in normal prostate and prostate cancer tissues [18]. The functional significance of the two *HNF1B* SNPs examined here is unknown, although a lymphocyte-derived gene expression analysis showed a significant association between rs4430796 and *HNF1B* expression in individuals of European ancestry but not in individuals of African ancestry [1].

The *G* allele of rs4430796 which is associated with decreased risk of endometrial cancer, has been associated with a decreased risk of prostate cancer but not with other cancers such as breast, lung, colorectal or pancreatic cancers or melanoma [2]. The same SNP allele has also been associated with an increased risk of type 2 diabetes [3], [4]. Diabetes is inversely associated with prostate cancer [19], but positively associated with endometrial cancer [20]. Therefore we may expect that SNPs would often have an effect in the same direction on both outcomes. The opposite effect of rs4430796 on diabetes and endometrial cancer, however, does not mirror the positive association between diabetes and endometrial cancer risk. We observed significant associations between *HNF1B* variants and endometrial cancer only among non-diabetics in both studies. The lack of statistical significance among diabetics is likely due to the small number of diabetics and thus limited power (<40%) in detecting modest effects associated with these SNPs. We also observed a potential interaction between *HNF1B* SNPs and diabetes status in the WHI, but not in the MEC. It is possible that this discrepancy was due to the fact that the magnitude of the association between diabetes and endometrial cancer differed between WHI (OR = 1.34; 95% CI: 0.93, 1.91) and MEC (OR = 0.93; 95% CI: 0.64, 1.36). In our analysis, adjusting for diabetes status had little effect on the SNP-endometrial cancer relationships. Whether diabetes status influences the association between *HNF1B* and endometrial cancer therefore remains unclear; examination of potential interaction between diabetes status and *HNF1B* in other endometrial cancer studies is warranted.

The strengths of our study include a relatively large sample size and the availability of comprehensive risk factor data for confounder adjustment, as well as an ancestrally diverse population. Limitations include non-centralized pathology review in determining the endometrial cancer histology which can result in misclassification of Type I and Type II tumors and can dilute the difference in ORs, if any, between these two groups.

In summary, we provide additional evidence that *HNF1B* is involved in endometrial cancer etiology. Future projects that include fine-mapping/sequencing the *HNF1B* region and functional studies are warranted to pinpoint the causal variants and the biological mechanisms involved in endometrial carcinogenesis.

## Supporting Information

Table S1 **Gene-environment interactions between** ***HNF1B*** **and endometrial cancer risk factors in the Women's Health Initiative Study (WHI).**
(DOCX)

Table S2 **Gene-environment interactions between** ***HNF1B*** **and endometrial cancer risk factors in the Multiethnic Cohort Study (MEC).**
(DOCX)
