# Supplementary material for: HNF1B and Endometrial Cancer Risk: Results from the PAGE study
Source: PLoS One. 2012 Jan 27;7(1):e30390. doi: 10.1371/journal.pone.0030390 (PMC3267708; doi:10.1371/journal.pone.0030390)
Supplement: Table S1 — Gene-environment interactions between HNF1B and endometrial cancer risk factors in the Women's Health Initiative Study (WHI). (DOCX) [file pone.0030390.s001.docx]

| Risk Factors |  | rs4430796 (*A/G*) |  |  | rs7501939 (*G/A*) |  |
| --- | --- | --- | --- | --- | --- | --- |
|  | Allele Frequency Cases/Controls | OR^1^ (95% CI) | P interaction* | Allele Frequency Cases/Controls | OR^1^ (95% CI) | P interaction* |
| Body Mass Index (kg/m^2^) |  |  |  |  |  |  |
| <25 | 0.44/0.48 | 0.83 (0.70, 0.99) | 0.29 | 0.33/0.40 | 0.72 (0.60, 0.87) | 0.10 |
| 25-<30 | 0.42/0.50 | 0.75 (0.61, 0.91) |  | 0.34/0.41 | 0.78 (0.64, 0.95) |  |
| 30+ | 0.49/0.52 | 0.91 (0.77, 1.08) |  | 0.40/0.44 | 0.85 (0.72, 1.02) |  |
| Parity |  |  |  |  |  |  |
| 0 | 0.45/0.48 | 0.88 (0.67, 1.13) | 0.67 | 0.38/0.41 | 0.87 (0.66, 1.15) | 0.57 |
| 1-2 | 0.44/0.51 | 0.75 (0.63, 0.90) |  | 0.33/0.42 | 0.67 (0.55, 0.80) |  |
| 3+ | 0.46/0.50 | 0.87 (0.75, 1.01) |  | 0.37/0.41 | 0.85 (0.73, 0.99) |  |
| Oral contraceptive use |  |  |  |  |  |  |
| Never | 0.45/0.51 | 0.80 (0.70, 0.91) | 0.28 | 0.36/0.42 | 0.78 (0.68, 0.90) | 0.85 |
| Ever | 0.46/0.49 | 0.90 (0.76, 1.06) |  | 0.35/0.40 | 0.80 (0.67, 0.95) |  |
| Menopausal hormone use^2^ |  |  |  |  |  |  |
| Never | 0.47/0.50 | 0.87 (0.74, 1.03) | 0.31 | 0.38/0.41 | 0.85 (0.71, 1.01) | 0.26 |
| Past | 0.41/0.51 | 0.67 (0.50, 0.90) |  | 0.31/0.43 | 0.57 (0.41, 0.78) |  |
| Current | 0.45/0.50 | 0.82 (0.69, 0.96) |  | 0.36/0.41 | 0.78 (0.65, 0.92) |  |
| Smoking status |  |  |  |  |  |  |
| Never | 0.46/0.49 | 0.87 (0.75, 1.00) | 0.60 | 0.38/0.41 | 0.87 (0.75, 1.01) | 0.15 |
| Past | 0.44/0.50 | 0.80 (0.68, 0.93) |  | 0.33/0.41 | 0.70 (0.59, 0.83) |  |
| Current | 0.50/0.53 | 0.94 (0.58, 1.51) |  | 0.38/0.42 | 0.84 (0.50, 1.37) |  |

^1^Odds ratio per allele obtained from logistic regression adjusting for age (continuous) and 4 ancestry principal components. Further adjusted for BMI for other risk factors.

^2^Also adjusted for clinical trial assignment.

*Test for interaction was assessed using log-likelihood test statistics comparing models with and without the interaction term.
