# Supplementary material for: HNF1B and Endometrial Cancer Risk: Results from the PAGE study
Source: PLoS One. 2012 Jan 27;7(1):e30390. doi: 10.1371/journal.pone.0030390 (PMC3267708; doi:10.1371/journal.pone.0030390)
Supplement: Table S2 — Gene-environment interactions between HNF1B and endometrial cancer risk factors in the Multiethnic Cohort Study (MEC). (DOCX) [file pone.0030390.s002.docx]

| Risk Factors |  | rs4430796 (*A/G*) |  |  | rs7501939 (*G/A*) |  |
| --- | --- | --- | --- | --- | --- | --- |
|  | Allele Frequency Cases/Controls | OR^1^ (95% CI) | P interaction* | Allele Frequency Cases/Controls | OR^1^ (95% CI) | P interaction* |
| Body Mass Index (kg/m^2^) |  |  |  |  |  |  |
| <25 | 0.42/0.43 | 0.92 (0.71, 1.20) | 0.29 | 0.33/0.37 | 0.77 (0.59, 1.02) | 0.74 |
| 25-<30 | 0.41/0.48 | 0.80 (0.60, 1.07) |  | 0.33/0.39 | 0.86 (0.64, 1.15) |  |
| 30+ | 0.39/0.50 | 0.68 (0.52, 0.88) |  | 0.33/0.40 | 0.78 (0.60, 1.02) |  |
| Parity |  |  |  |  |  |  |
| 0 | 0.43/0.47 | 0.92 (0.62, 1.35) | 0.71 | 0.32/0.37 | 0.81 (0.54, 1.22) | 0.40 |
| 1-2 | 0.38/0.47 | 0.71 (0.55, 0.92) |  | 0.30/0.38 | 0.69 (0.53, 0.91) |  |
| 3+ | 0.41/0.46 | 0.80 (0.64, 1.00) |  | 0.35/0.38 | 0.87 (0.69, 1.09) |  |
| Oral contraceptive use |  |  |  |  |  |  |
| Never | 0.40/0.45 | 0.79 (0.64, 0.97) | 0.58 | 0.33/0.37 | 0.83 (0.66, 1.03) | 0.96 |
| Ever | 0.43/0.47 | 0.84 (0.67, 1.06) |  | 0.35/0.39 | 0.82 (0.65, 1.04) |  |
| Menopausal hormone use^2^ |  |  |  |  |  |  |
| Never | 0.39/0.47 | 0.74 (0.57, 0.96) | 0.57 | 0.33/0.39 | 0.81 (0.62, 1.06) | 0.82 |
| Past | 0.46/0.48 | 0.95 (0.63, 1.44) |  | 0.38/0.38 | 0.92 (0.61, 1.39) |  |
| Current | 0.43/0.45 | 0.94 (0.68, 1.29) |  | 0.33/0.37 | 0.85 (0.61, 1.19) |  |
| Smoking status |  |  |  |  |  |  |
| Never | 0.39/0.45 | 0.78 (0.64, 0.94) | 0.40 | 0.33/0.37 | 0.83 (0.67, 1.01) | 0.27 |
| Past | 0.41/0.49 | 0.78 (0.59, 1.03) |  | 0.32/0.40 | 0.71 (0.53, 0.95) |  |
| Current | 0.52/0.48 | 1.07 (0.61, 1.88) |  | 0.43/0.40 | 1.10 (0.62, 1.96) |  |

^1^Odds ratio per allele obtained from logistic regression adjusting for age (continuous) and 4 ancestry principal components. Further adjusted for BMI for other risk factors.

^2^Limited to postmenopausal women.

*Test for interaction was assessed using log-likelihood test statistics comparing models with and without the interaction term.
